# A simplified checklist for the visual inspection of finished pharmaceutical products: a way to empower frontline health workers in the fight against poor-quality medicines

**DOI:** 10.1186/s40545-020-00211-9

**Published:** 2020-05-01

**Authors:** B. Schiavetti, E. Wynendaele, V. Melotte, J. Van der Elst, B. De Spiegeleer, R. Ravinetto

**Affiliations:** 1 Institute of Tropical Medicine Antwerp, Nationalestraat 155, B-2000 Antwerp, Belgium; 2 Department Pharmaceutical Analysis, DruQuaR (Drug Quality & Registration), Ghent University, Ottergemsesteenweg 460, B-9000 Ghent, Belgium; 3 Brussels, Belgium; 4 Centre for Adult Education CVO COOVI, Emile Grysonlaan 1, 1070 Brussels, Belgium; 5 Department of Public Health, Institute of Tropical Medicine Antwerp, Nationalestraat 155, B-2000 Antwerp, Belgium

**Keywords:** Medicines, Quality assurance, Developing countries, Visual inspection, Pharmacovigilance, Primary health care, Substandard, Falsified, Post-marketing surveillance, Point of care, Health workers

## Abstract

**Background:**

Substandard and falsified medicines, mainly prevalent in low and middle-income countries (LMICs), cause avoidable morbidity and mortality, and put at stake the performance of health systems. They may be prevented by an adequate implementation of pharmaceutical Quality Assurance (QA) guidelines, but unfortunately, most guidelines address upstream stakeholders and specialized staff in the supply chain. A multi-layered approach is needed, in order to empower the health workers at the point-of-care to proactively contribute to the fight against poor-quality medicines.

Visual inspection is a simple technique, suitable for field screening. The findings of a survey conducted in the Democratic Republic of the Congo (DRC) suggested that it might be a fairly good (yet partial) predictor of poor-quality, when compared to full laboratory tests.

**Methods and results:**

Starting from the 68-questions checklist originally used in the survey in the DRC, we developed a simplified checklist, specifically designed to guide health workers at the point of care to rapidly identify suspect poor-quality medicines. We selected those medicines’ attributes the assessment of which does not require technical expertise, or access to regulatory information. Attributes were categorized according to a 3-level risk scale, to guide decision-making on suspect poor-quality medicines, based on an informed risk assessment.

The simplified checklist contains 26 binary questions (YES/NO), grouped into four themes: packaging, identification, traceability, and physical appearance. Each non-conformity corresponds to a level of risk for patients. The user is guided towards three possible actions: A) reasonably safe for dispensing; B) dispense with explanation; C) quarantine and make a risk-benefit evaluation before dispensing.

**Conclusion:**

The simplified checklist should now be implemented in real-life setting in LMICs. If proven useful in guiding health workers at the point-of-care to take rapid, transparent, patient-centred actions when facing a suspect poor-quality medicine, it could be further extended to address specific formulations. Digitalization for linkage with pharmacovigilance programs could also be considered.

## Introduction

Poor-quality medicines hamper the effectiveness of health systems in low and middle-income countries (LMICs) [1–3]. They include substandard medicines, caused by poor manufacturing or distribution practices, and falsifications, prompted by a deliberate intent to fraud [4]. The circulation of poor-quality medicines is favoured by the weakness of National Medicines Regulatory Authorities (NMRAs) and the poor traceability of intermediate and finished pharmaceutical products along the supply chains [5]. In 2017, the World Health Organization Global Surveillance and Monitoring System (WHO-GSMS) estimated that 10,5% of medicines in LMICs are of poor-quality [6].

Pharmaceutical quality assurance (QA) guidelines, generally address stakeholders upstream in the supply chain (manufacturers, distributors) and specialists (qualified persons, inspectors, pharmacists) [7, 8]. However, in LMICs, poor-quality medicines can reach hospitals and health centres where specialists are rare or absent [6]. Therefore, to decrease the risk that poor-quality medicines reach the patients, and to strengthen the credibility of health systems, it is important to raise the awareness of healthcare workers at the point of care, and to empower them to detect and manage suspect poor-quality products [6].

Various field-screening tools have been developed in recent years, but they are generally unsuitable for use at the point of care: they require ad hoc training and access to technical information, and they can be costly and product- or group-specific [9, 10]. The World Health Profession Alliance published the Be Aware tool for visual inspection, and the International Pharmaceutical Federation developed a similar tool [11, 12]. Both checklists, though, address mainly falsifications, and require access to/understanding of regulatory or technical information. Therefore, we developed a simplified visual inspection checklist, to allow field screening by healthcare workers at the point of care.

## Methods and results

We previously conducted a survey to assess the quality of a sample of medicines available in the Democratic Republic of Congo [13]. Medicines underwent visual inspection, carried out locally by inspectors of the NMRA, and physico-chemical analysis, conducted at accredited Quality Control laboratories. To carry out the visual inspection, we developed a 68-questions standardized checklist for tablets and powders for suspension. The choice of the 68 attributes to inspect was guided by the requirements of the International Pharmacopoeia (Ph.Int) [14]. Although we did not calculate the statistical correlation between the visual non-conformities and the results of physico-chemical analysis, 75,5% of the samples that failed laboratory tests was also non-conform for some attributes of the physical appearance. This checklist was developed for research purposes, which justifies its length and complexity (68 questions and 5 themes). However, a simpler tool would be required for use in routine surveillance by non-specialists.

Therefore, in order to make it suitable for use by health workers at the point of care, we developed a shorter and simplified 26-questions checklist. To select the questions to retain, we used the following criteria:

- Questions on medicines’ attributes that can be checked by non-specialists
- Questions on medicines’ attributes that can be checked without accessing complementary technical or regulatory information

For instance, the question “conformity of the marketing authorisation” and the verification of the stated manufacturer were excluded, because they would require retrieving regulatory information. Similarly, the adequacy of the information leaflet was excluded, because assessing it would require technical expertise. Out of the 68 binary questions (YES/NO) and five themes of the original checklist, 23 questions were retained and grouped into four themes: A. Packaging, B. Identification, C. Traceability, D. Physical appearance.

In May 2019, a draft version of the simplified checklist was pre-tested with pharmacists and nurses of five hospitals in the DRC. Based on their feedback, we added four questions on the attributes of sterile liquids and powders for injections, since health workers reported that these formulations are often problematic and that assessing their quality is a priority. We also adapted the questions about physical appearance of powders for suspension, in order to allow examining syrups as well; and we refined some wording to make them more user-friendly, e.g. we replaced the terms primary/secondary packaging with internal/external packaging. We excluded one question on residual powder in the blisters of tablets, because this can be a consequence of tablets cracking (already included in the checklist), and is not mentioned in the International Pharmacopoeia.

**Fig. 1:**
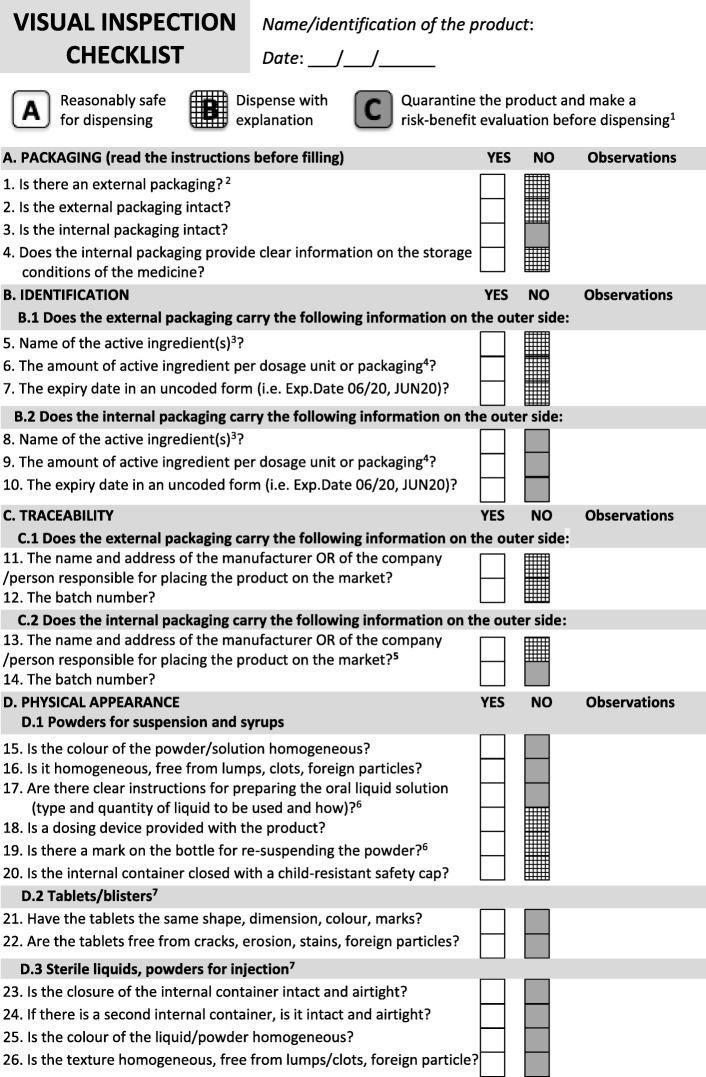
Visual Inspection Checklist (new version)

**Fig. 2:**
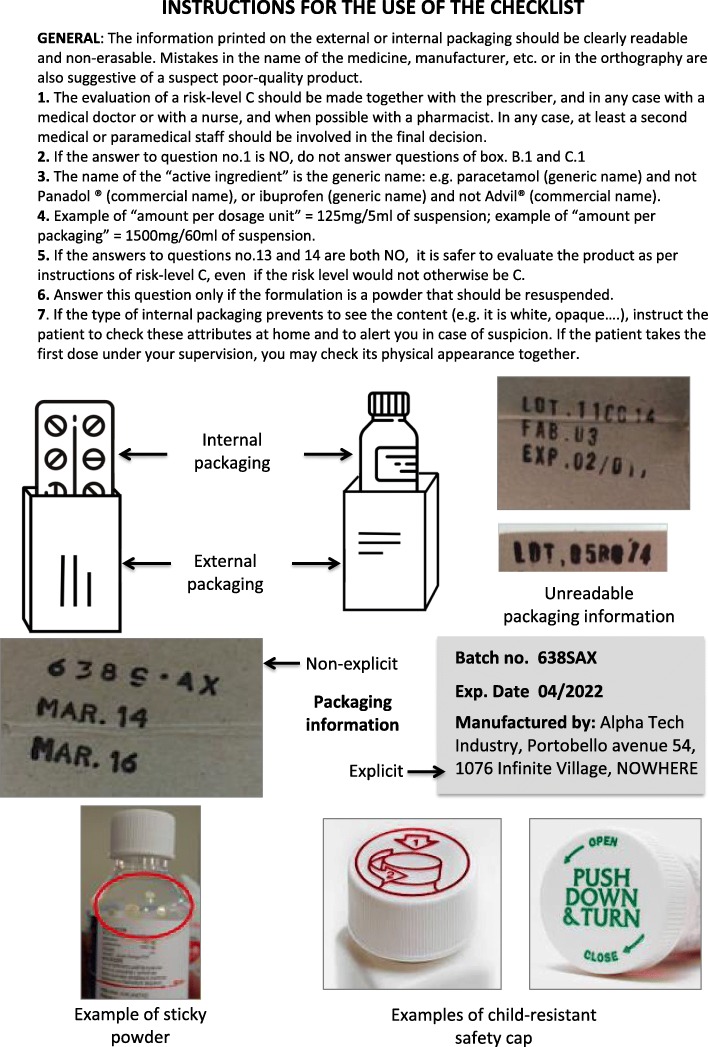
Instruction for the use of the checklist

The final checklist contains 26 questions covering the most common formulations, so as to allow the healthcare workers to assess most of the medicines they handle (Fig. 1). Instructions for use of the checklist are attached (Fig. 2).

For each attribute/question, we considered the “level of risk”, i.e. if/to what extent a non-conformity would be harmful for the patient. Taking into account the principles of the WHO QA guidelines for pharmaceuticals [15], as well as the authors first-hand experience, we identified a three-levels risk-scale (A-B-C), where each non-conformity corresponds to a different kind of action to manage suspect poor-quality medicines identified through the checklist:

- *A) Reasonably safe for dispensing:* no visible non-conformity has been identified. The medicine can be dispensed.
- *B) Dispense with explanation*: some non-conformities have been identified, but they are thought not to compromise the safety or efficacy of the medicine.
  This is the case if, for instance, some key-attributes required for the identification, conservation, or traceability of the medicine are missing on the secondary packaging; or, if the secondary packaging is damaged; or if, (for multi-dose preparations) a child-resistant closure or an appropriate dosing device are lacking [16]. The medicine can only be dispensed after carefully instructing the patient or caregiver on its storage conditions, preparation and administration (as applicable).
  Conversely, if the attributes for traceability of the product are lacking on the internal packaging (Fig. 1, Questions 13 and 14), than the risk level increases from B to C, as this is a critical non-conformity.
- C) *Quarantine and make a risk-benefit evaluation before dispensing*: some non-conformities have been identified, and they can surely compromise the safety or efficacy of the medicine.
  This is the case, for instance, if the primary (internal) packaging is damaged; or, if key-information for the identification, shelf life or preparation of the suspension is missing; or, if there are visible non-conformities (i.e. clumps, stickiness, heterogeneous colour, residual powder). The medicine should be quarantined, and the prescriber informed.
  A risk-based assessment should be done with the prescriber or, in any case, with a medical doctor. Ideally, a pharmacist should be involved in the decision-making process as the product may need to be replaced, e.g. when the primary packaging is damaged. However, pharmacists may not be present in remote health posts in LMICs, thus this role will be shifted to the concerned healthcare provider and his/her supervisor.
  If it is not possible to (rapidly) replace the suspect poor-quality product, an informed decision must be made on a case-by-case basis (e.g. *Is it a life-saving treatment? Can an alternative treatment be prescribed?*). The case should be reported to the persons responsible for the health post, for due follow-up with the supplier, and with regulatory authorities as needed.

## Discussion and conclusion

Although poor physical appearance does not necessarily correlate with poor physico-chemical quality, it is an important attribute of the quality assurance of medicines and it is included in the International Pharmacopoeia. In addition, it may be a fairly good predictor of chemical non-conformities. For instance, in the quality survey we previously conducted in the DRC, 75,5% of the anti-malarial powders for suspensions that were found to be under strength with regard to artemether content, had also visible physical non-conformities.

In resource-limited settings, visible non-conformities confront health workers with multifaceted questions and sometimes with ethical dilemma: should the product be discarded, or dispensed? In the former case, there may be a risk of leaving the patient untreated, due to unavailability of an alternative medicine. In the latter case, there is a risk of harming the patient, since the visual non-conformity can be the indicator of poor physico-chemical quality. Full laboratory tests can be performed for additional investigations, but they are costly and time-consuming, and not fit to guide rapid decisions.

Conversely, a simple, standardized visual inspection checklist is a low-cost, low-training, frugal technique that could be implemented as routine practice. Even if it cannot rule out all non-conformities, it covers most formulations included in the WHO Model List of Essential Medicines, both for adults and children [17, 18], and can orient health workers at the point of care to make rapid, informed and patient-centred decisions, when facing suspect poor-quality medicines. In LICs, where Pharmacy and Therapeutic Committees are rarely functional, the use of a structured checklist may also foster multidisciplinary collaboration among healthcare workers; promote transparency and accountability in the health system, given that decisions will be reasoned, informed and documented; and increase the awareness of poor-quality medicines among health workers at the point of care, who are unlikely to be reached otherwise by specific trainings.

Overall, we hope that a simple checklist has the potential to empower frontline health workers to become active stakeholders in the detection of poor-quality medicines [19]. On a side note, the checklist is also suitable to assess medicines in bulk packs, before repackaging (which must comply with Good Pharmacy practices) [20].

In general, even if it is required by WHO QA guidelines as part of a functioning quality system, the visual inspection of medicines presents some important limitations: non-visible non-conformities will go undetected, and the assessment may also be biased by subjective evaluation. Therefore, it is important that its value is well understood, and not overestimated, by the users and by their supervisors. Furthermore, the use of the checklist needs to be modelled according to each country’s specificities. For instance, responsibilities to conduct the risk-benefit assessment in case of risk-level C, should be assigned according to the local organizational structure and to the competences of the personnel at different levels of the healthcare pyramid.

The checklist proposed here is mainly relevant for low-income countries with weak regulatory authorities, reported high prevalence of poor-quality medicines, limited resources for rapidly replacing dubious batches, and a dearth of pharmacists, especially in remote health facilities. However, some middle-income countries might find it useful at peripheral level.

We hope that this tool may contribute to making visual inspection a routine practice at the “last mile” of the supply chain. As next steps, it should be implemented in real-life settings, and adapted as needed to the challenges and specific regulatory features of each context. If proven user-friendly and effective, it could be extended to different formulations (topical, etc.); and digitalization for linkage with pharmacovigilance programs could be considered.

## Supplementary information

**Additional file 1.** Visual Inspection Checklist (previously submitted version)

## Data Availability

The initial 68-questions checklist is provided as additional documentation. The manuscript results from the follow-up work to a medicines quality survey conducted in the DRC: Schiavetti B, Wynendaele E, De Spiegeleer B, Mbinze GJ, Kalenda N, Marini R, et al. The Quality of Medicines Used in Children and Supplied by Private Pharmaceutical Wholesalers in Kinshasa, Democratic Republic of Congo: A Prospective Survey. Am J Trop Med Hyg. 2018;98 (3):894–903. 10.4269/ajtmh.17-0732

## References

[1] World Health Organization. A study on the public health and socioeconomic impact of substandard and falsified medical products. Geneva; 2017. WHO/EMP/RHT/2017.02. Available: https://www.who.int/medicines/regulation/ssffc/publications/se-study-sf/en/. Accessed 20 Apr 2020.

[2] Newton PN, Caillet C, Guerin PJ (2016). A link between poor quality antimalarials and malaria drug resistance?. Expert Rev Anti-Infect Ther.

[3] Ozawa S, Evans DR, Bessias S, Haynie DG, Yemeke TT, Laing SK (2018). Prevalence and estimated economic burden of substandard and falsified medicines in low- and middle-income countries. JAMA Netw Open.

[4] World Health Organization. (22–31 May 2017). Seventieth World Health Assembly. Annex A70/23. Appendix 3. Geneva, Switzerland. Available: https://www.who.int/medicines/regulation/ssffc/A70_23-en1.pdf?ua=1. Accessed 2 Sept 2019.

[5] World Health Organization. Norms and standards. 70 years of WHO standards on medicines quality. Expert committee on specifications for pharmaceutical preparations, 1947-2017: addressing changing public health challenges. Who Drug Info 2017. 2017;31(1):15–26. Available: https://www.who.int/medicines/publications/druginformation/issues/WHO_DI_31-1_Norms-Standards.pdf?ua=1. Accessed 20 Apr 2020.

[6] World Health Organization. WHO Global Surveillance and Monitoring System for substandard and falsified medical products. Geneva: World Health Organization; 2017. Licence: CC BY-NC-SA 3.0 IGO. Available: https://www.who.int/medicines/regulation/ssffc/publications/gsms-report-sf/en/ Accessed 20 Apr 2020.

[7] World Health Organization. A Model quality assurance system for procurement agencies. In: Annex III of the WHO technical report series 986: WHO Expert Committee on Specifications for Pharmaceutical Preparations, forty-eighth report. Geneva. Available: https://www.who.int/medicines/areas/quality_safety/quality_assurance/expert_committee/ISBN9789241209861-TRS986.pdf. Accessed 20 Apr 2020.

[8] Hamilton WL, Doyle C, Halliwell-Ewen M, Lambert G (2016). Public health interventions to protect against falsified medicines: a systematic review of international, national and local policies. Health Policy Plan.

[9] S, Fernandez FM, Newton PN, Caillet C. Field detection devices for screening the quality of medicines: a systematic review. BMJ Glob Health. 2018;3:e000725. 10.1136/bmjgh-2018-000725 PMC6135480 30233826

[10] Kovacs S, Hawes SE, Maley SN, Mosites E, Wong L (2014). Technologies for detecting falsified and substandard drugs in low and middle-income countries. Plos One.

[11] World Health Professions Alliance. Be Aware: Tool for visual inspection of medicines; 2011. p. 4. Available: https://www.whpa.org/sites/default/files/2018-12/Toolkit_BeAware_Inspection.pdf. Accessed 20 Apr 2020.

[12] International Pharmaceutical Federation, United States Pharmacopeia. A checklist for visual inspection of medicines. Available: https://www.fip.org/files/fip/counterfeit/VisualInspection/A%20tool%20for%20visual%20inspection%20of%20medicines%20EN.pdf. Accessed 20 Apr 2020.

[13] Schiavetti B, Wynendaele E, De Spiegeleer B, Mbinze GJ, Kalenda N, Marini R, et al. The quality of medicines used in children and supplied by private pharmaceutical wholesalers in kinshasa, democratic Republic of Congo: a prospective survey. Am J Trop Med Hyg. 2018;98(3):894–903. Available: 10.4269/ajtmh.17-0732. Accessed 20 Apr 2020. 10.4269/ajtmh.17-0732 PMC5930909 29313479

[14] World Health Organization. The International Pharmacopoeia. 6th ed; 2016. Available: https://apps.who.int/phint/2016/index.html#p/home. Accessed 20 Apr 2020.

[15] World Health Organization. Quality Assurance of pharmaceuticals, WHO guidelines, good practices, related regulatory guidance and GXP training materials. 2016. Available: http://digicollection.org/whoqapharm/p/about/. Accessed 20 Apr 2020.

[16] World Health Organization. Guidelines on packaging for pharmaceutical products. WHO Tech Rep Ser. 2002;902(902):120–54. Available from: https://apps.who.int/iris/bitstream/handle/10665/42424/WHO_TRS_902.pdf. Accessed 20 Apr 2020.

[17] World Health Organization (2019). Model List of Essential Medicines, 21st List, 2019.

[18] World Health Organization (2019). Model List of Essential Medicines for Children, 7th List, 2019.

[19] Ravinetto R, Pinxten W, Rago L (2018). Quality of medicines in resource-limited settings: need for ethical guidance. Global Bioethics.

[20] Lehmann A, et al. Drug Quality In South Africa: A Field Test. J Pharm Sci. 2018. 10.1016/j.xphs.2018.06.012 [Epub ahead of print]. 10.1016/j.xphs.2018.06.012 29936204

