# Supplementary material for: A simplified checklist for the visual inspection of finished pharmaceutical products: a way to empower frontline health workers in the fight against poor-quality medicines
Source: J Pharm Policy Pract. 2020 May 1;13:9. doi: 10.1186/s40545-020-00211-9 (PMC7193355; doi:10.1186/s40545-020-00211-9)

Fig1

**VISUAL INSPECTION CHECKLIST**

(for powders for suspensions and tablets)

| **A. PACKAGING (see instructions, before filling)** | **YES** | **NO** | **Observation** |
| --- | --- | --- | --- |
| 1. Is there an external packaging^3^? |  |  |  |
| 2. Is the external packaging intact? |  |  |  |
| 3. Is the internal packaging intact? |  |  |  |
| 4. Does the internal packaging provide clear information on the storage conditions of the medicine? |  |  |  |
| **B. IDENTIFICATION** | **YES** | **NO** | **Observation** |
| ***B.1 Does the external packaging carry the following information on the outer side:*** | | | |
| 5. Name of the active ingredient(s)^4^? |  |  |  |
| 6. The amount of active ingredient per dosage unit or packaging^5^? |  |  |  |
| 7. The expiry date in an uncoded form (i.e. 06/2020, 06/20, JUN20)? |  |  |  |
| ***B.2 Does the internal packaging carry the following information:*** | | | |
| 8. Name of the active ingredient(s)^4^? |  |  |  |
| 9. The amount of active ingredient per dosage unit or packaging^6^? |  |  |  |
| 10. The expiry date in an uncoded form (i.e. 06/2020, 06/20, JUN20)? |  |  |  |
| **C. TRACEABILITY** | **YES** | **NO** | **Observations** |
| ***C.1 Does the external packaging carry the following information on the outer side:*** | | | |
| 11. The name and address of the manufacturer OR of the company  /person responsible for placing the product on the market? |  |  |  |
| 12. The batch number? |  |  |  |
| ***C.2 Does the internal packaging*** ***carry the following information:*** | | | |
| 13. The name and address of the manufacturer OR of the company  /person responsible for placing the product on the market? |  |  |  |
| 14. The batch number? |  |  |  |
| **D. PHYSICAL APPEARANCE** | **YES** | **NO** | **Observations** |
| ***D.1 Powders for suspension*** | | | |
| 15. Is the colour of the powder homogeneous? |  |  |  |
| 16. Is the powder free from lumps, stickiness or dampness? |  |  |  |
| 17. Are there clear instructions for preparing the oral liquid solution (type and quantity of liquid to be used, and how)? |  |  |  |
| 18. Is a dosing device provided with the product? |  |  |  |
| 19. Is there a mark on the bottle for re-suspending the powder? |  |  |  |
| 20. Is the internal container closed with a child-resistant safety cap? |  |  |  |
| ***D.2 Tablets/blisters^7^*** | | | |
| 21. Have the tablets the same shape, dimension, colour, marks? |  |  |  |
| 22. Are the tablets free from cracks, erosion, stains, foreign particles, and visible traces of contamination? |  |  |  |

Quarantine the product and make a risk-benefit evaluation before dispensing^2^


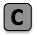


Dispense with explanation^1^


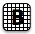


Reasonably safe for dispensing


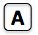

Supplement: Supplementary file 1 — Additional file 1. Visual Inspection Checklist (previously submitted version) [file 40545_2020_211_MOESM1_ESM.docx]
